# Dietary supplementation of inulin alleviates metabolism disorders in gestational diabetes mellitus mice via RENT/AKT/IRS/GLUT4 pathway

**DOI:** 10.1186/s13098-021-00768-8

**Published:** 2021-12-24

**Authors:** Miao Miao, Yongmei Dai, Can Rui, Yuru Fan, Xinyan Wang, Chong Fan, Juan Mu, Wenwen Hou, Zhiyong Dong, Ping Li, Guiju Sun, Xin Zeng

**Affiliations:** 1 Nanjing Maternity and Child Health Care Hospital, Women’s Hospital of Nanjing Medical University, 210004 Nanjing, Jiangsu P. R. China; 2 Key Laboratory of Environmental Medicine and Engineering of Ministry of Education, Department of Nutrition and Food Hygiene, School of Public Health, Southeast University, 210004 Nanjing, Jiangsu P. R. China

**Keywords:** Gestational diabetes, Fasting blood glucose, Inulin, Glycolysis/gluconeogenesis pathway, RETN

## Abstract

**Background:**

Gestational diabetes mellitus (GDM) has significant short and long-term health consequences for both the mother and child. There is limited but suggestive evidence that inulin could improve glucose tolerance during pregnancy. This study assessed the effect of inulin on glucose homeostasis and elucidated the molecular mechanisms underlying the inulin-induced antidiabetic effects during pregnancy.

**Method:**

Female C57BL/6 mice were randomized to receive either no treatment, high-dose inulin and low-dose inulin for 7 weeks with measurement of biochemical profiles. A real-time^2^ (RT^2^) profiler polymerase chain reaction (PCR) array involved in glycolipid metabolism was measured.

**Results:**

Inulin treatment facilitated glucose homeostasis in a dose-dependent manner by decreasing fasting blood glucose, advanced glycation end products and total cholesterol, and improving glucose tolerance. Suppressing resistin (RETN) expression was observed in the inulin treatment group and the expression was significantly correlated with fasting blood glucose levels. The ratios of p-IRS to IRS and p-Akt to Akt in liver tissue and the ratio of p-Akt to Akt in adipose tissue as well as the expression level of GLUT4 increased significantly after inulin treatment.

**Conclusions:**

Our findings indicated improvement of glucose and lipid metabolism by inulin was to activate glucose transport through the translocation of GLUT4 which was mediated by insulin signaling pathway repairment due to decreased expression of RETN and enhanced phosphorylation of IRS and Akt in GDM mice.

**Supplementary Information:**

The online version contains supplementary material available at 10.1186/s13098-021-00768-8.

## Introduction

Gestational diabetes mellitus (GDM)-defined as hyperglycemia, insulin resistance, and carbohydrate intolerance with the onset or first recognition during pregnancy-is a common obstetric complication, affecting an estimated 15.8% of pregnancies worldwide [1, 2]. GDM women have an increased risk of adverse maternal and perinatal complications, including preeclampsia, hydramnios, increased operative intervention and future type 2 diabetes mellitus, macrosomia [3, 4], congenital anomalies, metabolic abnormalities [5–9], and subsequent childhood and adolescent obesity [10]. In addition, increased levels of inflammatory mediators and biomarkers of oxidative stress can induce maternal insulin resistance, DNA damage, and chromosomal aberrations [11, 12].

This perpetuates an intergenerational cycle of disease that further escalates the obesity epidemic. To break this cycle, it would be beneficial to generate therapies that prevent GDM from developing [13]. Current treatments include diet and lifestyle interventions, followed by insulin treatment and, in some countries, oral agents such as metformin. Although women are able to maintain adequate glycemic control using these treatment strategies, they can be difficult to implement, and concerns remain regarding the long-term effects of oral agents on the developing fetus. For these reasons, it would be beneficial to develop novel, safe, and effective strategies for GDM risk reduction [14].

Inulin is a soluble dietary fiber, which is stored in the tubers of a perennial herb Jerusalem artichoke [15, 16]. Due to the specific chemical structure, inulin dietary fiber is not digested in the human mouth, stomach, or small intestine, so that it does not cause increase in blood glucose concentration, but rather prevents sharp changes in blood sugar and protecting islet cells after ingestion, thus known as “natural insulin” for diabetic patients [17]. In 2017, a systematic review of clinical trial results showed that dietary supplementation with inulin reduced biomarkers of metabolic syndrome [18]. Misiakiewicz-Has et al. found supplementation with inulin had a negative effect on plasma glucose in both diabetic and non-diabetic rats [19]. In the United States in 2018, the Food and Drug Administration approved inulin as a dietary fiber ingredient used to improve the nutritional value of manufactured food products. Inulin-treated hyperglycemic mice had decreased average daily food consumption, body weight, average daily water consumption and relative liver weight and blood concentrations of TAG, total cholesterol, HDL-cholesterol and fasting blood glucose[20].

Furthermore, inulin has been associated with improved glucose metabolism and reduced risk of GDM [21]. While the mechanisms linking inulin to metabolic health are poorly understood, inulin are known to modify the intestinal microbiome and stimulate production of short-chain fatty acids (SCFAs). SCFAs affect the expression of a number of proteins that have been demonstrated to decrease gut permeability and increase insulin sensitivity [22, 23]. However, the evidence that inulin supplementation should be recommended before or during pregnancy to reduce the risk of GDM is limited [24, 25]. This study was, therefore, carried out to investigate the mechanism and effects of inulin supplementation on glycolipid metabolism and pregnancy outcomes in GDM mice.

## Materials and methods

### Animals and study design

Six to seven-week-old C57BL/6J mice were purchased from Vital River Laboratory Animal Technology Co., Ltd., (China) and acclimatized to the animal facility for 1 week. Mice were maintained on a 12-h light-dark cycle with free access to food and water. All experiments were performed in accordance with the Animals (Scientific Procedures) Act 1986 and approved by the Ethics Committee of Women’s Hospital of Nanjing Medical University (No.2018-49).

Female mice were fed normal chow diet (NCD group, Research Diets AIN-93G, consisting of 20.3% protein, 63.9% carbohydrate, and 15.8% fat), a high-fat/sucrose diet (HFD) (GDM group, Research Diets D12451, consisting of 19.8% protein, 35.2% carbohydrate, and 45% fat), high-dose inulin supplemented a high-fat/sucrose diet (Inulin-H group, 3.33 g/kg/days via oral gavage ) or low-dose inulin supplemented a high-fat/sucrose diet (Inulin-L group, 1.67 g/kg/days via oral gavage) for 4 weeks before being mated with age-matched male mice. Upon identification of a copulatory plug, considered to be day 0 of pregnancy (GD0). Mice were euthanized by CO_2_ inhalation on GD18 (or equivalent) after fasting for 6 h from 8 AM and blood and tissues were collected. Liver and inguinal fat was quickly collected and kept at − 80 °C.

### Measurement of body weight, blood glucose and serum insulin

Body weight, blood glucose and serum insulin were monitored at different time points, including before dietary intervention, after 4 weeks of HFD, and on GD 0, 10, 14 and 18. Blood glucose and insulin levels were determined from tail venipuncture blood samples. Blood glucose concentration was measured immediately using a blood glucose meter and strips (Roche Accu-Chek Active, Mannheim, Germany). The blood samples were then centrifuged at low speed (4 °C, 3000 rpm, 15 min) within 1 h, the supernatant was harvested and stored at − 80 °C for measuring serum insulin level by enzyme linked immune sorbent assay (ELISA; NJJCBIO Co., Ltd, Nanjing, China) according to the manufacturer’s instructions. The homeostasis model assessment of insulin resistance (HOMA-IR) was calculated by the following formula: fasting blood glucose (FBG, mmol/L) × fasting serum insulin (µIU/mL)/ 22.5. In addition, urine volume and fluid intake were observed daily.

### Oral glucose tolerance test

Glucose tolerance was determined by an oral glucose tolerance test (OGTT) on GD 14. Mice were fasted for 6 h with free access to water and received an oral gavage of 20% D-glucose (2 g/kg body weight). Blood samples were collected from the tail vein at 0, 30, 60, 90 and 120 min after glucose administration. Blood glucose levels were measured instantly using methods as mentioned above. Meanwhile, area under the curve (AUC) of blood glucose was calculated [26].

### Serum lipid measurement and total AGEs

Levels of serum triglycerides (TG), total cholesterol (TC), low-density lipoprotein (LDL) and high-density lipoprotein (HDL) as well as the concentration of Serum total advanced glycation end products (AGEs) were measured using commercial kits (NJJCBIO Co., Ltd, Nanjing, China) according to the manufacturer’s instructions.

### Haematoxylin-eosin staining

Liver and inguinal fat tissues were fixed in 4% paraformaldehyde, decalcified, paraffin-embedded and stored at 4 °C. After tissues were sliced into 4 μm sections, haematoxylin-eosin staining was performed. First, sections were stained with haematoxylin for 5–10 min, immersed in 70% ethanol for 30 min to remove cytoplasm colouring, alkalized with alkaline solution and washed with distilled water for 1 min. Second, sections were stained with eosin for 30–60 s, dehydrated with gradient ethanol, cleared two times with xylene, dried and mounted. Finally, the morphological structures of the liver and inguinal fat tissues were observed under an optical microscope.

### RT^2^ profiler PCR array analysis

Total RNA was isolated from the liver samples of NCD group, GDM group and Inulin-H group using Qiagen RNeasy® Mini Kit (QIAGEN, Shanghai, China) according to the manufacturer’s instructions. Single-strand cDNA was synthesized from 1 µg of total RNA by reverse transcription reaction using Qiagen RT^2^ First Strand Kit (QIAGEN, Shanghai, China). The cDNA was mixed with Qiagen PCR RT^2^ SYBR Green Master Mix (QIAGEN, Shanghai, China).

To explore the underlying mechanisms of inulin induced-effects, the expression of 84 genes involved in Glycolipid metabolism including RETN were examined using RT^2^ profiler PCR array (PAMM-006Z-mouse glucose metabolism, QIAGEN, Shanghai, China). Relative quantification of mRNA levels was determined by real-time quantitative PCR using a ABI 7500 RT-PCR machine/Bio-Rad CFX96 Sequence Detector instrument. The quantitative expression of gene was calculated from the cycle threshold (CT) value of each sample in the linear part of the curve using the relative quantification method (2^−ΔΔCT^) [27]. The samples were analyzed in triplicate and corrected for the selected internal standard which had the smallest standard deviation among the housekeeping genes. Candidate genes were selected from those whose expressions differed greater than twofold or less than twofold, or which differed significantly (*p* < 0.05) between the GDM group and Inulin-H treatment group.

### Quantitative PCR (qPCR) analysis for the candidate genes

The five most differentially-expressed genes between GDM group and Inulin-H group were selected from the RT^2^ profiler PCR array as candidate genes and were analyzed the correlation with FBG further. Specific PCR primers were designed for further quantitative real-time PCR analysis (Takara, Dalian, China) as follows.

G6pc: 5’-CGACTCGCTATCTCCAAGTGA-3’ and 5’-GGGCGTTGTCCAAACAGAAT-3’.

RETN: 5’-ACAAGACTTCAACTCCCTGTTT-3’Cand 5’-TTTCTTCACGAATGTCCCACG-3’.

Igfbp5: 5’-CCCTGCGACGAGAAAGCTC-3’ and 5’-GCTCTTTTCGTTGAGGCAAACC-3’.

Slc14a2: 5’-AAGGAGATGTCTGACAGCAACA-3’ and 5’-GGGCTGGGTGTGTATCCTG-3’.

IL10: 5’-CCCATTCCTCGTCACGATCTC-3’and 5’-TCAGACTGGTTTGGGATAGGTTT-3’.

### Western blot analysis

Liver and inguinal fat tissues were harvested and were homogenized on ice in the presence of protease and phosphatase inhibitors. Homogenates were centrifuged at 12,000×*g* at 4 °C for 15 min. Protein concentration in supernatants was quantified by the BCA method using bovine serum albumin (BSA) as the standard. Proteins were analyzed by 10% SDS-PAGE and transferred to PVDF membranes that were incubated in 5% non-fat milk at room temperature for 1 h, then incubated with appropriate primary and secondary antibodies: Phospho-IRS-1 (Ser1101) antibody (#2385T, 1:1000 dilution for WB), IRS-1 (D23G12) antibody (#3407T, 1:1000 dilution for WB), p44/42 MAPK (Erk1/2) (137F5) antibody (#4695T, 1:1000 dilution for WB), Phospho-p44/42 MAPK (Thr202/Tyr204) antibody (#4370T, 1:1000 dilution for WB), Akt (pan) antibody (#4685, 1:1000 dilution for WB) and Phospho-Akt (Ser473) antibody (#4060, 1:1000 dilution for WB) were obtained from Cell Signaling Technology, Inc. (MA, USA). Resistin (ab119501, 1:1000 dilution for WB) and GLUT4 antibody (ab33780, 1:1000 dilution for WB) was purchased from Abcam (MA, USA). β-actin (20536-1-AP, 1:5000 dilution for WB) and HRP-conjugated Affinipure Goat Anti-Rabbit (SA00001-2, 1:10,000 for WB) were purchased from Proteintech Group, Inc (IL, USA). Color prestained protein Marker (180-6003) was purchased from Tanon Science and Technology Co., Ltd. (Shanghai, China). Membranes were washed and proteins were detected by enhanced chemiluminescence (ECL) using a LAS-4000 lumino-image analyzer (Fuji Film, Tokyo, Japan). Bands were digitally scanned and analyzed using ImageJ software (NIH Image, National Institutes of Health, Bethesda, MD, USA).

### Statistical analysis

All data were calculated as means ± SD and checked using the Kolmogorov-Smirnov (KS) test before further analysis. Statistical significance between two datasets was assessed using the Student’s t-test. Multiple groups were compared using one-way ANOVA followed by Tukey multiple comparison testing. For the repeated measures in case of growth and OGTT results, multivariate ANOVA was performed with a post hoc test using Bonferroni method. A *P* value of < 0.05 was considered statistically significant. All statistical tests were performed using GraphPad Prism Version 7.0 (GraphPad Prism Software, Inc. CA, USA).

## Result

### Changes of body weight and reproductive outcome of pregnant mice

**Fig. 1:**
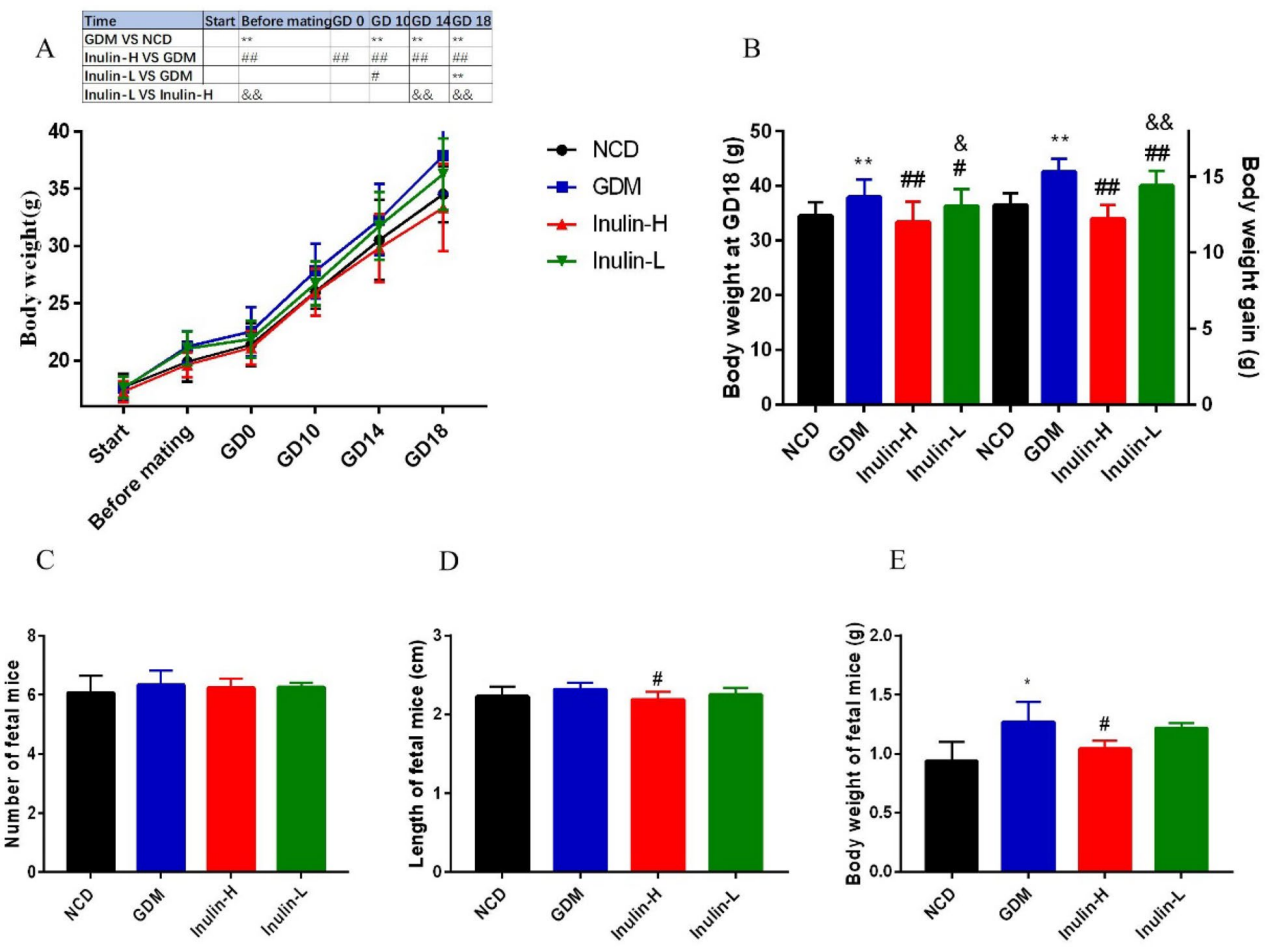
Changes of body weight (**A**, **B**), the number, length of fetal mice (**C**, **D**) and body weight at birth (**E**). Data (n = 15) are expressed as the mean ± SD values and analysed by one-way or multivariate ANOVA followed by Tukey’s or Bonferroni’s post-tests: *P <0.05, **P<0.01 vs. NCD group; #*P* <0.05, ##*P* <0.01 vs. GDM; &*P* <0.05, &&*P* <0.01 vs. Inulin-H group

Body weight was determined at different time points for all groups. As indicated in Fig. 1A, the body weight of GDM group showed an increasing trend after 4 weeks of HFD prior to mating, and a rapid elevation of body weight was found in the mice of GDM group compared to the moderate increase in NCD group and Inulin-H group during peripartum. The weight at GD 18 and total weight gain of GDM (37.88±3.32 g and 15.32±0.86 g) and Inulin-L group (36.28±3.16 g and 14.39±0.97 g) were significantly higher than that of NCD group (34.54±2.42 g and 13.13±0.78 g) and Inulin-H group (33.34±3.80 g and 12.22±0.92 g) (Fig. 1B). After inulin intervention, the Inulin-H group showed no statistically significant difference in body weight compared with the NCD group (Fig. 1A, B). In addition, the urine volume and fluid intake were increased significantly during mid-to-late pregnancy in GDM mice compared to NCD mice. However, there was no significant change in the mice of Inulin-L or Inulin- H group.

The number, body weight and length of fetal mice in each group were compared. There was no significant difference in the number of fetal mice among groups (Fig. 1C). The average body weight and length of fetal mice born by GDM mothers (1.27±0.17 g and 2.32±0.08 cm) were significantly higher than those by NCD (0.94±0.16 g and 2.23±0.12 cm )and Inulin- H mothers (1.04±0.07 g and 2.19±0.10 cm) (Fig. 1D, E).

### Glucose tolerance test, blood glucose, serum insulin and total AGEs in mice

**Fig. 2:**
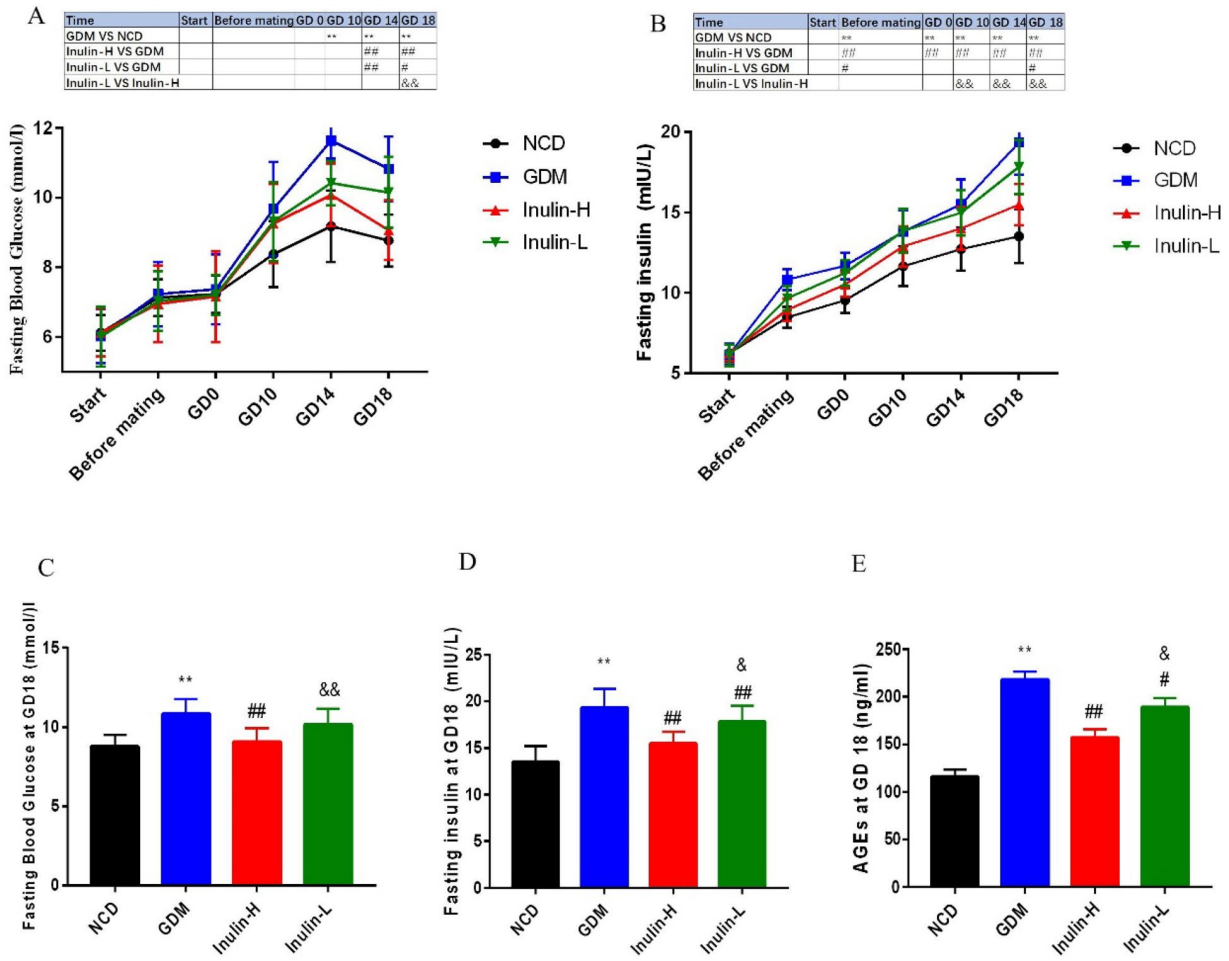
Changes of blood glucose (**A**, **C**), and serum insulin (**B**, **D**) in mice of different groups before dietary intervention, after 4 weeks of HFD, and on GD 0, 10, 14 and 18. AGE’s (**E**) on GD 18. Data(n = 15) are expressed as the mean ± SD values and analysed by one-way or multivariate ANOVA followed by Tukey’s or Bonferroni’s post-tests: **P<0.01 vs. NCD group; ##*P* <0.01 vs. GDM; &*P* <0.05, &&*P* <0.01 vs. Inulin-H group

We examined the blood glucose and serum insulin in these mice. Blood glucose levels did not differ amongst the groups at the end of 4-week feeding before mating and exhibited a gradual upregulation during pregnancy in the mice of GDM and Inulin-L groups but not NCD or Inulin-H group (Fig. 2A). Moreover, blood glucose, serum insulin and total AGEs on GD 18 were significantly advanced in GDM mice (10.83±0.93 mmol/l, 19.34±1.98 mIU/l and 218±8.6 ng/ml) in contrast to the NCD mice (8.77±0.75 mmol/l, 13.52±1.68 mIU/L and 116±7.8 ng/ml ) (Fig. 2C–E). Interestingly, blood glucose levels were deregulated at the end of pregnancy in each group (Fig. 2A), whereas insulin levels were increased in both GDM and Inulin-L mice and showed a more pronounced enhancement in the mice of GDM group (Fig. 2B). After inulin intervention, the Inulin-H group showed no statistically significant difference in blood glucose, insulin and total AGEs changes compared with the NCD group (Fig. 2C–E).

**Fig. 3:**
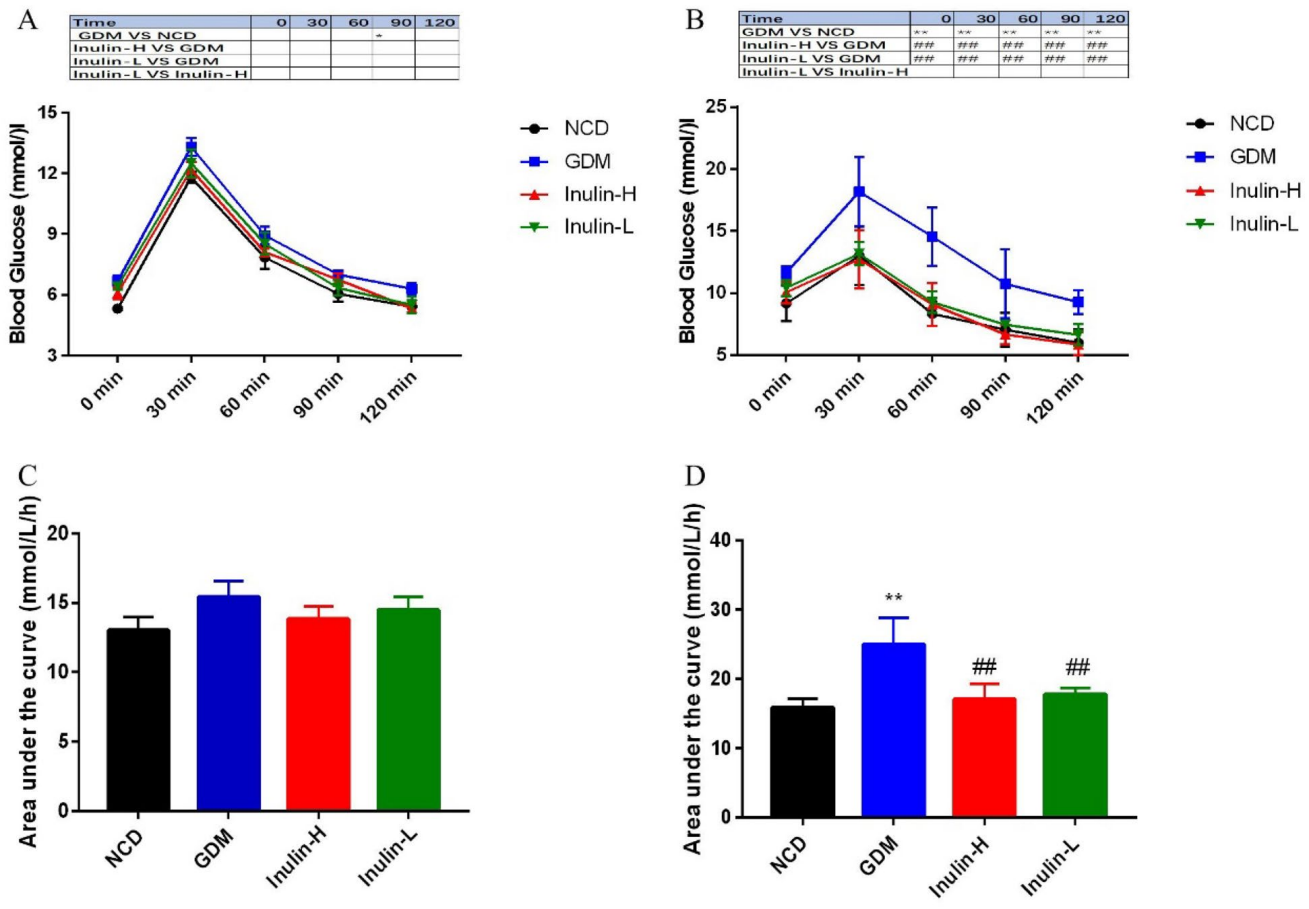
Blood glucose (**A**, **C**) during oral glucose tolerance test in the four groups after 4 weeks of HFD. Blood glucose (**B**, **D**) during oral glucose tolerance test in the four groups on GD 14. Data (n = 15)are expressed as the mean ± SD values and analysed by one-way or multivariate ANOVA followed by Tukey’s or Bonferroni’s post-tests: **P<0.01 vs. NCD group; ##*P* <0.01 vs. GDM

By performing a glucose tolerance test at the end of 4-week feeding before mating, we found that blood glucose and insulin levels of mice in GDM group indicated a slight increase without statistically significant differences, suggesting HFD for 4 weeks did not cause dramatical changes in either glucose or insulin levels (Fig. 3A, C). Then, we conducted the same test on GD 14. As shown in Fig. 3B, compared with NCD group, the mice in GDM group showed impaired glucose tolerance, as manifested by obviously increased glucose levels after glucose injection (*P* < 0.01, 0, 30, 60, 90 and 120 min). While the blood glucose of Inulin-L and Inulin- H group mice were dramatically lower than those of GDM group throughout the test (*P* < 0.01, Fig. 2G), as were the AUCs (Fig. 3D). All these suggested that inulin treatment could significantly improve the glucose tolerance of GDM mice.

### Effects of inulin on lipid profiles and tissues morphology in mice

**Fig. 4:**
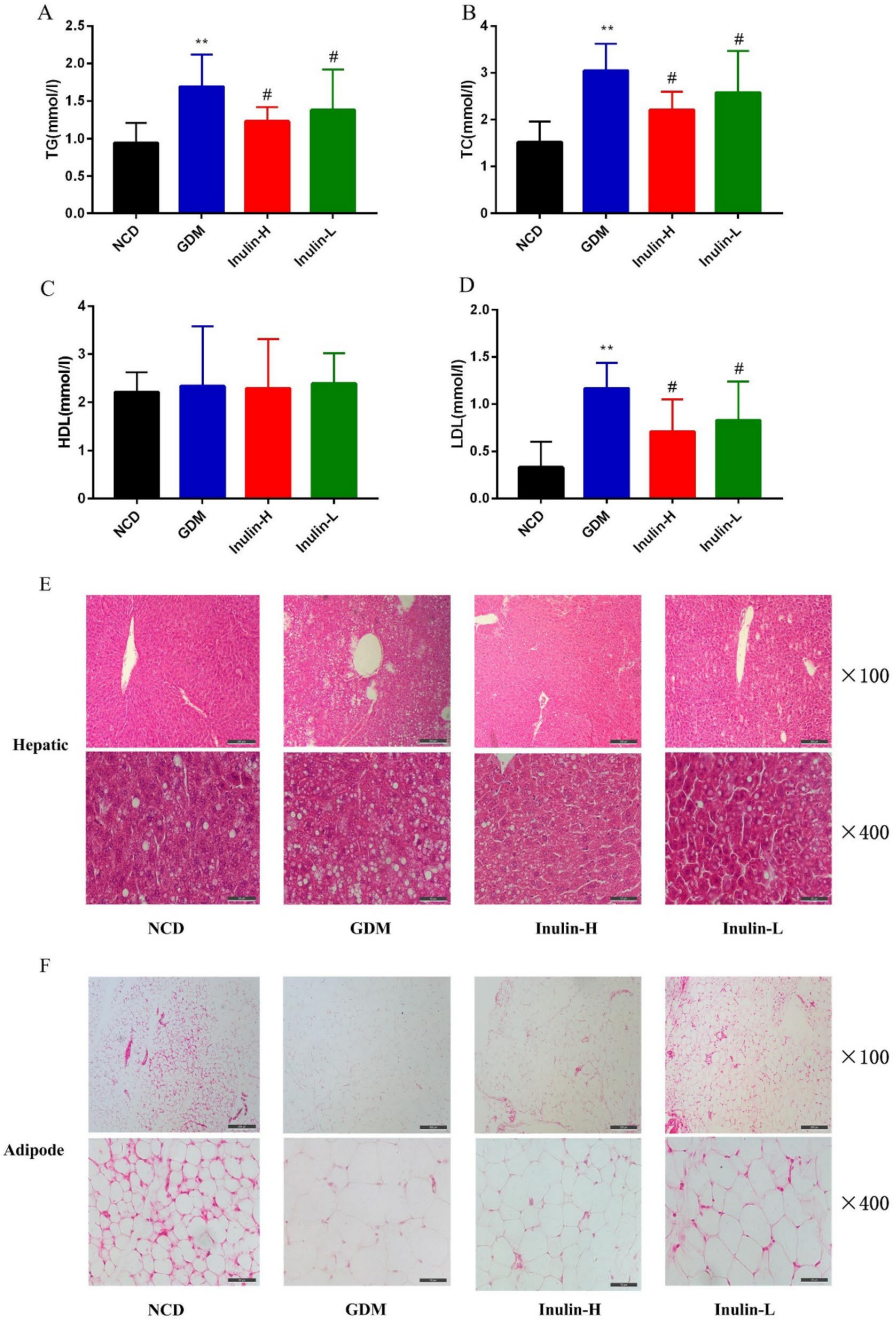
Changes of lipid prolife (**A**-**D**) in mice of different groups on GD 18. Data (n = 15) are expressed as the mean ± SD values and analysed by one-way followed by Tukey’s post-tests: **P<0.01 vs NCD group; #*P* < 0.05 vs GDM. Changes of hepatic and adipose morphology (**E**, **F**). The hepatic and adipose tissues were stained with HE for morphologic analysis under light microscopy. Morphologic changes of hepatic and adipose cells were observed under at different magnifications field (× 100 and × 400)

The levels of TG, TG and LDL in GDM group were significantly higher than those in the other three groups, suggesting that HFD could elevate serum lipid levels of GDM mice, while inulin intervention could significantly reduce serum triglyceride, total cholesterol and low-density lipoprotein levels of GDM mice (Fig. 4A, B, D). However, there was no significant difference in serum HDL among groups (Fig. 4C).

This finding was supported by histology, which showed an increase in lipid droplets in hepatic and adipose tissues from mice fed the HFD diet and the HFD diet supplemented with inulin compared to NCD. Hepatic lipid droplets and adipocyte hypertrophy were significantly alleviated in mice from Inulin-L and Inulin- H group compared to GDM group (Fig. 4E and F), suggesting that inulin could attenuate the fat accumulation induced by HFD.

### Effects of inulin on the expression of those genes involved in glycolipid metabolism pathway

**Fig. 5:**
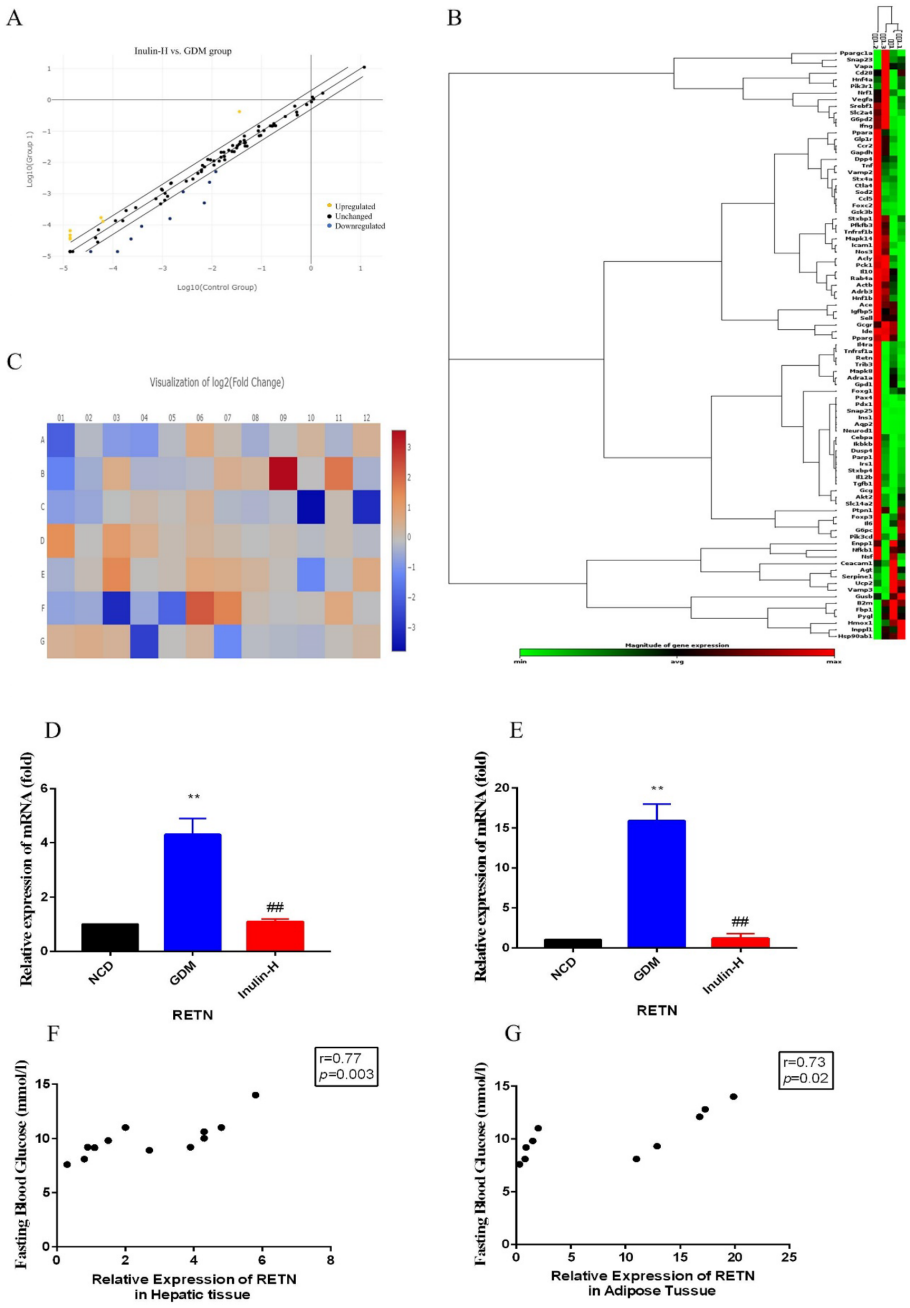
Changes of the candidate genes expressions between GDM and Inulin-H groups by using RT^2^ profiler PCR array. **A** Scatter plot of the results of every gene expression. The central diagonal line indicates unchanged gene expression, while the outer diagonal lines indicate the selected fold regulation threshold. Yellow dots denote genes with upregulated expression, while blue dots represent genes with downregulated expression. Black dots denote genes with unchanged expression. **B** Cluster gram of genes with significantly upregulated or downregulated expression. Each column expresses a group, and each row represents a gene. Various colors display the magnitude of gene expression and the degree of gene expression from minimum to maximum is exhibited from green to red. **C** Heat map of the results of every gene expression. Different colors show the different levels of gene expression. Colors ranging from blue to red means downregulated to upregulated gene expression. The most differentially-expressed gene (RETN) validated with qPCR in hepatic (**D**) and adipose tissues (**E**). Correlation between RETN expression in hepatic (**F**) and adipose tissues (**G**) and fasting glucose levels. Differences were assessed by t-test or Wilcoxon rank-sum test, as appropriate. Correlation analysis was performed with Pearson pairwise test. **P < 0.01 vs. NCD group; ##*P* < 0.01 vs. GDM

Sixteen genes involved in the glycolipid metabolism were detected with different expressions between Inulin-H group mice and GDM mice in hepatic tissues (Fig. 5A–C). The five most differentially-expressed genes (G6pc, Slc14a2, Igfbp5, RETN, Il10) were validated by qPCR with the specific primers in hepatic and adipose tissues. We found that the 5 five genes’ expressions were consistent with RT^2^ profiler PCR array results, and the consistent results were observed on the housekeeping genes as well. In particular, the expression of RETN was significantly down-regulated after inulin treatment compared to GDM in hepatic (1.10±0.01 vs 4.30±0.60) (Fig. 5D) and adipose tissues (1.17±0.01 vs15.86±2.10) (Fig. 5E). We next examined the association between RETN relative expression in hepatic and adipose tissues and FBG levels on GD18. We found that increased RETN expression was significantly correlated with increasing fasting glucose (Fig. 5F, G).

### Measurement of alterations of protein expression by western blotting

Previous studies have shown that RETN played an important role in the regulation of insulin signaling pathway. RETN could induce insulin resistance by inhibiting the phosphorylation of IRS, Akt and ERK 1/2 proteins [28].

**Fig. 6:**
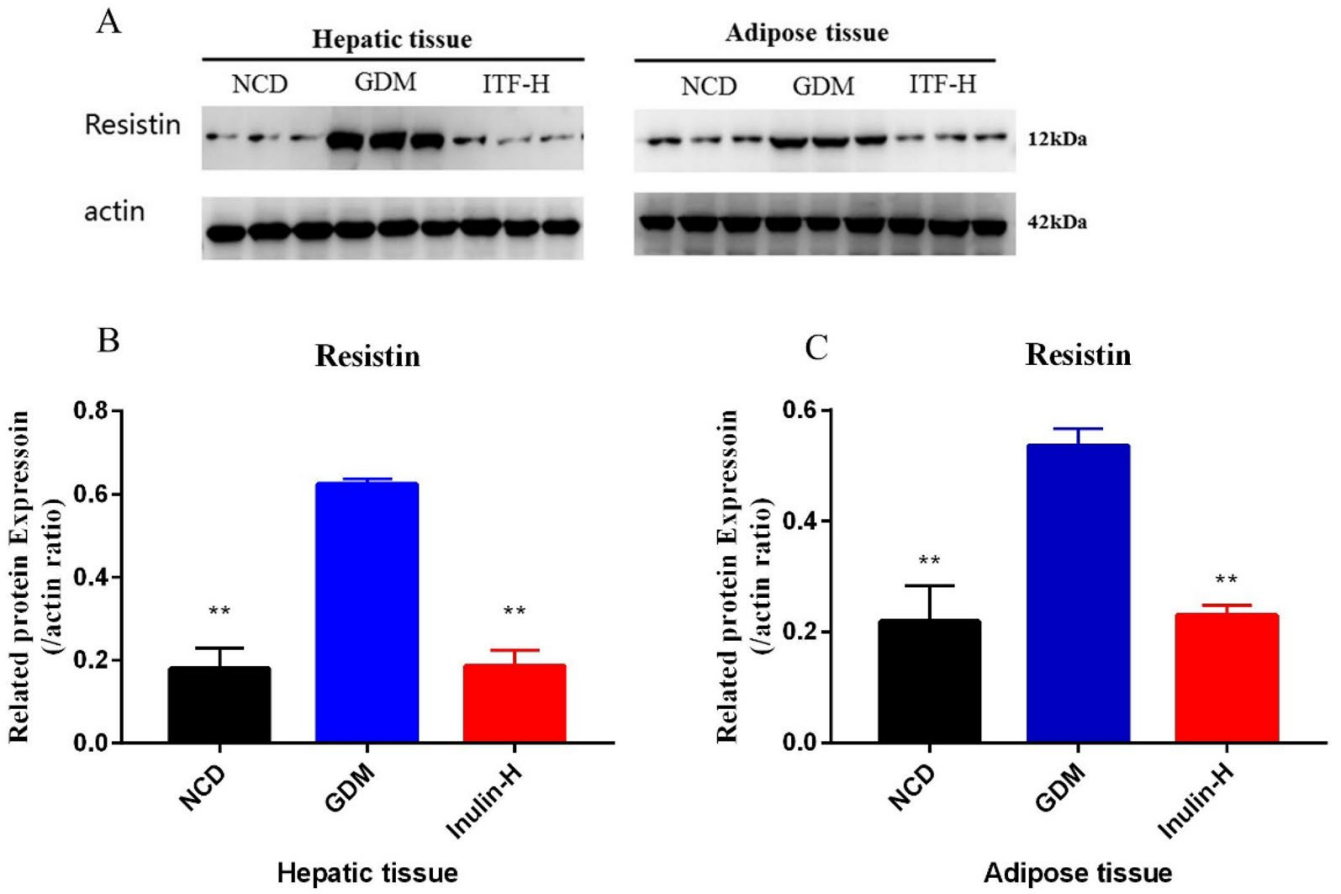
Changes of hepatic and adipose RETN protein expression levels. Representative immunoblot (**A**) and quantification of RETN (**B**,** C**) in the hepatic and adipose tissues of mice from the three groups. Data (n = 15) are expressed as the mean ± SD values and analysed by one-way followed by Tukey’s post-tests: **P < 0.01 vs. NCD group; ##*P* <0.01 vs. GDM

**Fig. 7:**
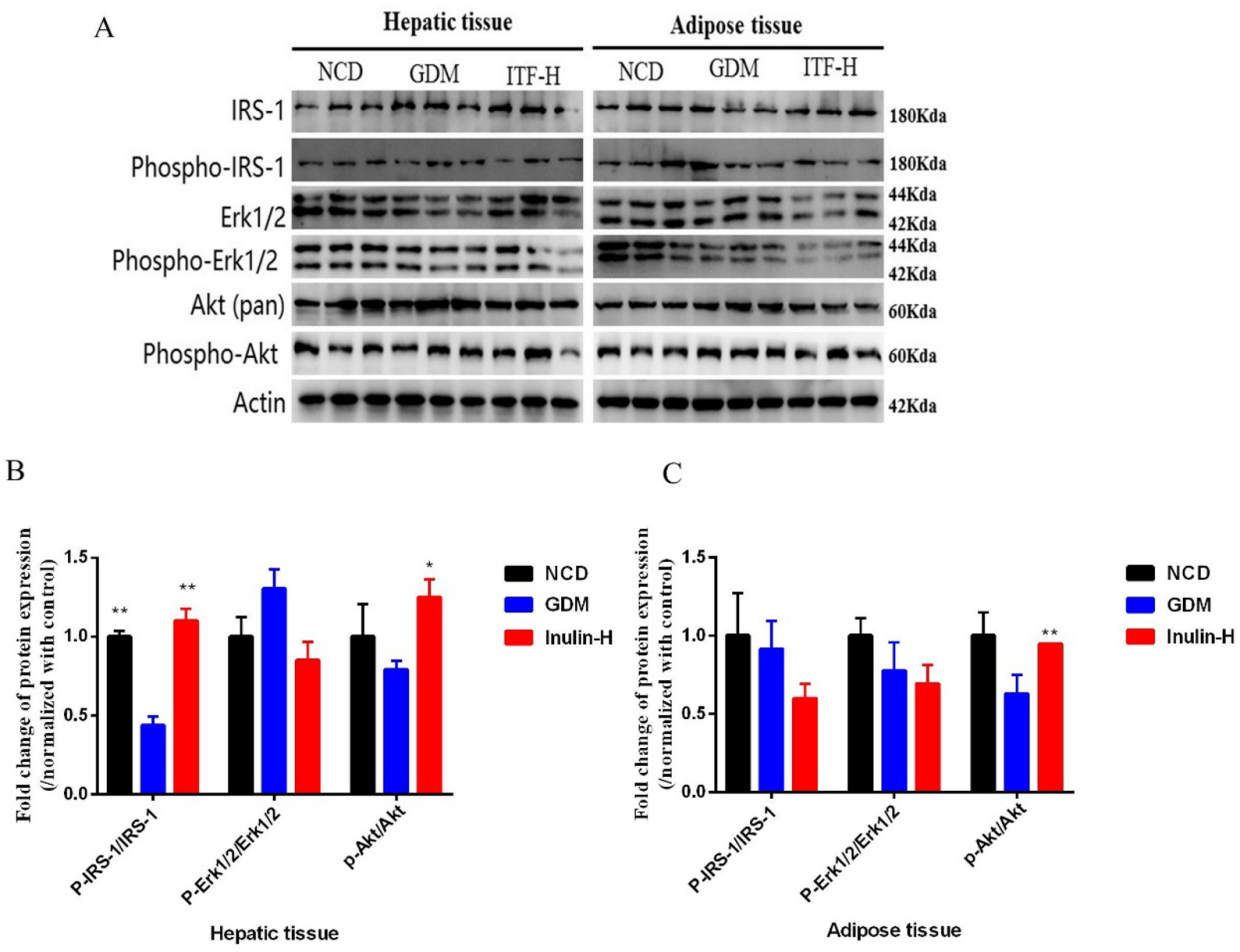
Changes of hepatic and adipose IRS-1, Erk and Akt protein expression levels. Representative immunoblot of IRS-1, p-IRS-1, Erk, p-Erk, Akt and p-Akt in the hepatic and adipose tissues of mice from the three groups (**A**). The ratios of p-IRS to IRS, Erk to p-Erk and Akt to p-Akt in the hepatic (**B**) and adipose tissues (**C**) of mice from the three groups. Data (n=15) are expressed as the mean ± SD values and analysed by one-way followed by Tukey’s post-tests: **P<0.01 vs. NCD group; #*P* <0.05, ##*P* <0.01 vs. GDM

**Fig. 8:**
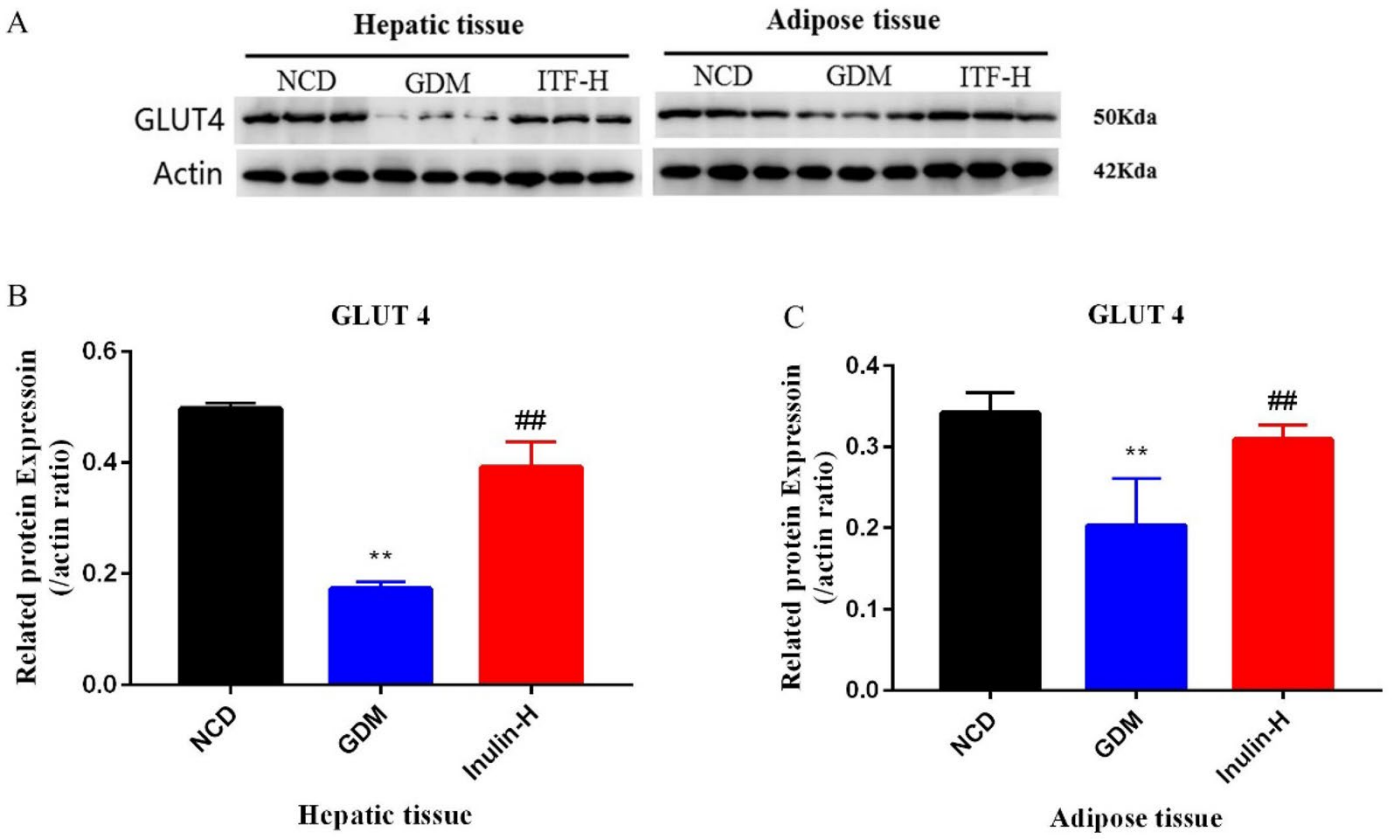
Changes of hepatic and adipose GLUT4 protein expression levels. Representative immunoblot (**A**) and quantification of GLUT4 (**B**, **C**) in the hepatic and adipose tissues of mice from the three groups. Data (n = 15) are expressed as the mean ± SD values and analysed by one-way followed by Tukey’s post-tests: **P<0.01 vs. NCD group; ##*P* <0.01 vs. GDM

Western blotting was used to investigate any changes of RETN and these kinases (IRS, Akt and ERK 1/2) after inulin treatment in mouse liver and adipose tissue. The western blot analysis results showed that the protein level of RETN was consistent with the mRNA results (Fig. 6A–C). The ratio of p-IRS to IRS signal molecule in liver tissue of GDM group was significantly reduced compared to NCD group (Fig. 7A, B). After the ITF-H treatment, the ratios of p-IRS to IRS and p-Akt to Akt in liver tissue and the ratio of p-Akt to Akt in adipose tissue increased significantly (Fig. 7 A, C). Similarly, it was demonstrated that administration of inulin has increased the protein level of GLUT4 (Fig. 8A–C). These results suggested that inulin could enhance the phosphorylation of IRS and Akt signaling molecules and accelerate GLUT 4 translocation to improve glucose and lipid metabolism in GDM mice.

## Discussion

The recommended diet for glycemic control should be rich in dietary fiber [29]. Inulin is a widely distributed carbohydrate in nature, which is a recognized dietary fiber [30]. In the present study, inulin improved glucose tolerance, reduced insulin resistance, and lowered body weight, serum total AGEs, serum triglyceride, total cholesterol and low-density lipoprotein levels in GDM mice. Moreover, inulin improved the pregnancy outcomes of GDM mice. In addition, a further significant decrease of RETN expression and increase of the phosphorylation level of IRS and Akt signaling molecules in insulin signaling pathway, thereby the increase of GLUT4 expression were observed upon exposure to inulin. Taken together, our study demonstrates a beneficial effect of inulin in the control of gestational diabetes, and this effect is related to the activation of insulin signaling pathway.

GDM is a metabolic disorder that is characterized by hyperglycemia accompanied with insulin resistance. In the present study, the blood glucose level and insulin level were significantly increased in the GDM group mice on GD 18, indicating that GDM was successfully established in these animals [31, 32]. AGEs have been implicated in the pathogenesis of both type 1 and type 2 diabetes [33]. Several studies have shown that AGEs are associated with insulin resistance [34, 35], and can induce low-grade inflammation [36] and pancreatic beta cell dysfunction [37]. Serum total AGEs on GD 18 were significantly advanced in GDM mice. We also observed disorders of lipid metabolism in GDM mice as evidenced by elevated serum levels of TG, TC and LDL, which is similar to the metabolic profile in the humans with GDM [38]. The therapeutic effects of inulin on metabolic disorders have been extensively reported [39–41]. A previous study showed that oral administration of inulin at 5 g/kg/day for 10 weeks protected the rats from diabetes-induced renal injury [42]. In the present study, we chose the doses of 3.33 and 1.67 g/kg/day to evaluate the potential antidiabetic effects of inulin in mice. The results showed that 4 weeks before mating and throughout pregnancy treatment with inulin relieved the diabetic symptoms as evidenced by reduced blood glucose level and insulin level. However, Farhangi et al. found that inulin significantly reduced the fasting serum glucose level and HbA1C ratio, but had little effect on the insulin level in patients with T2DM [43]. Our results showed that inulin modulated glucose metabolism in a dose-dependent manner, thus we speculate that the differential effects of inulin on insulin may be due to different dosages (10 g/day for T2DM patients in Farhangi et al.’s study). Moreover, we observed a strong hypolipidemic effect of inulin in GDM rats. These results agree with a previous study showing that inulin promoted lipid metabolism by altering the expression of acetyl-CoA carboxylase and the activities of fatty acid synthase and xanthine oxidase [40].

Liver and adipose are major contributors that responsible for lipid and glucose metabolism, and they are sensitive to insulin signal for the regulation of glucose homeostasis [44]. In the pathological conditions of GDM, insulin action is compromised and glucose uptake by these cells were disrupted, resulting in hyperglycemia [45, 46]. In the present study, treatment with chicory inulin could attenuate increased lipid droplet size in retroperitoneal fat tissue and hepatic lipid accumulation induced by HFD.

**Fig. 9:**
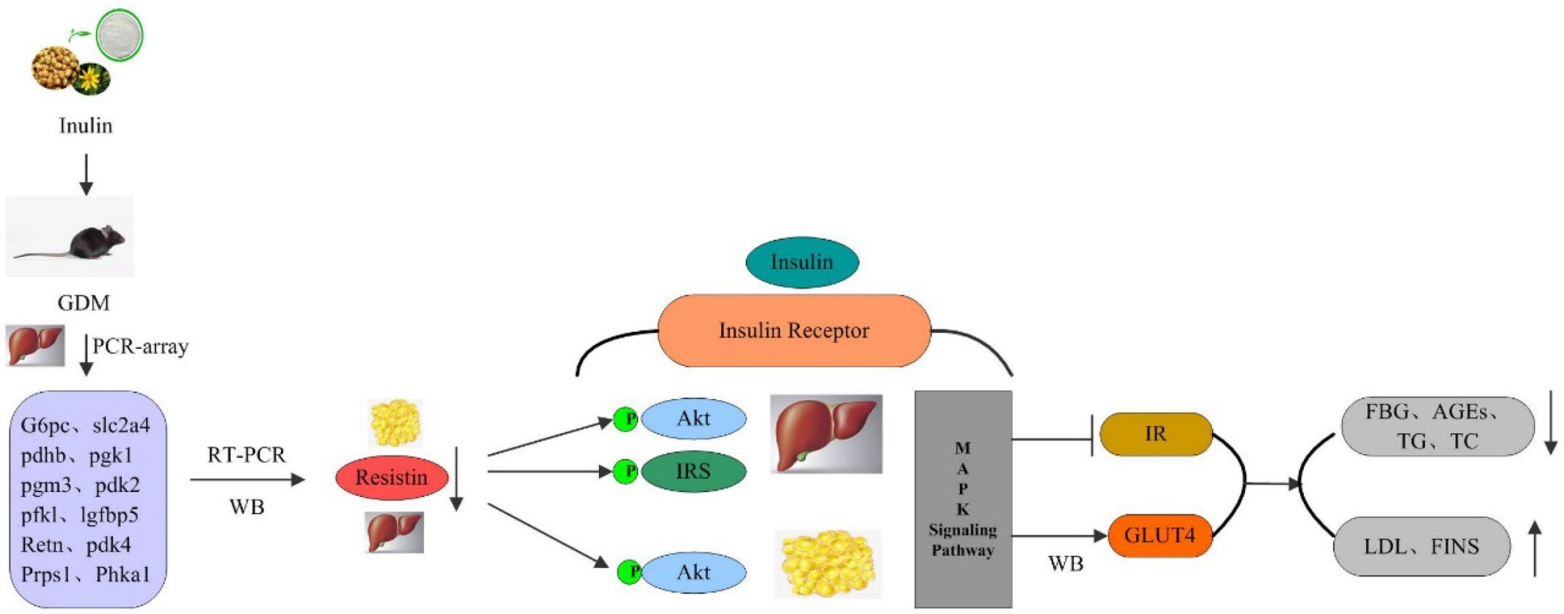
Presumed mechanism by which inulin alleviated glucose and lipid metabolism disorders in gestational diabetes mellitus mice. The mechanism underlying improvement of glucose and lipid metabolism by inulin was to activate glucose transport through the translocation of GLUT4 which was mediated by insulin signaling pathway repairment due to decreased expression of RETN and enhanced phosphorylation of IRS and Akt in GDM mice. Finally, this signal leads to changes in downstream metabolic functions. GDM: Gestational diabetes mellitus; FBG: Fasting blood glucose; IR: Insulin resistance; AGEs: Advanced glycation end products; TG: Triglycerides; TC: Total cholesterol; LDL: Low-density lipoprotein; HDL: High-density lipoprotein; RETN: Resistin; IRS: Insulin receptor; Akt: Protein kinase B; ERK 1/2: Extracellular signal-regulated protein kinases 1 and 2; GLUT4: Glucose transporter isoform 4

To explore the underlying mechanisms of inulin induced antidiabetic effects, the expression of 84 genes involved in glucose metabolism were examined. Five candidate genes with most differential-expression following inulin treatment were validated, and they were mainly involved in the glycolysis/gluconeogenesis pathway and participated in different types of metabolism process including glucose metabolic process and gluconeogenesis. Insulin signaling plays a pivotal role in maintaining basic cellular functions such as synthesis and degradation of glycogen, lipids and proteins [47]. In this study, we found that RETN expression was the most down-regulated after inulin treatment, and this down-regulation was positive correlated with fasting glucose level in GDM mice. RETN is a pro-inflammatory adipokine that as an influential factor interferes with insulin action, signaling, and glucose metabolism [47–50]. Benomar et al. demonstrated that RETN interfered with normal insulin signaling pathway by inhibiting phosphorylation of IRS (the major downstream target of insulin receptor), Akt, and ERK 1/2 [28]. GLUT4 is the main insulin-responsive glucose transporter which was cloned and studied in several laboratories in 1989. Those studies showed that GLUT4 proteins are translocated to plasma membrane by the action of insulin through the insulin signaling pathway [51]. In this present study, the ratios of p-IRS to IRS and p-Akt to Akt in liver tissue and the ratio of p-Akt to Akt in adipose tissue as well as the expression level of GLUT4 increased significantly after the inulin treatment. The mechanism underlying improvement of glucose and lipid metabolism by inulin was to activate glucose transport through the translocation of GLUT4 which was mediated by insulin signaling pathway repairment due to decreased expression of RETN and enhanced phosphorylation of IRS and Akt in GDM mice (Fig. 9).

The molecular systems assessed in the current study are at the critical interface between cell metabolism; thereby having a strong impact on other physiological parameters, which suggests that insulin can influence a range of cellular processes. In agreement, a large body of literature suggests that many food-derived bioactive compounds, such as Raffinose, aspalathin, curcumin and resveratrol, can positively affect glucose or lipid metabolism, in part by controlling major cellular response mechanisms such as activation of insulin dependent or independent signaling pathways in conditions of metabolic stress [52–55]. This study lays an important foundation for future investigations to inform how the dietary fiber, inulin, modulates the essential genes involved in energy regulation to enhance improve substrate metabolism (the uptake of glucose and palmitate) and enhance ATP (adenosine triphosphate) production in vitro.

In addition to RETN and insulin signaling pathway, some other genes and signaling pathways are also implicated in the regulation of glucolipid metabolism, such as AMP-activated protein kinase (AMPK) and PI3K/AKT pathways [56, 57]. Exploring whether exerts its glucose-control effects through these pathways, though beyond the scope of our present study, will provide interesting insights into the molecular mechanisms underlying inulin’s hypoglycemic action. Thus, further studies are needed to verify the involvement of other pathways in mediating the anti-diabetic actions of inulin.

## Conclusions

In summary, our study demonstrates that inulin could lower blood glucose level and improve glucolipid metabolism. Inulin treatment was associated with reduction in improvement of impaired oral glucose and insulin tolerance, lipid profile, liver structure and function, and it could also promote pregnancy outcomes in gestational diabetic mice. These actions are likely to be mediated via repairment of insulin signaling pathway. Our findings suggest a potential value of inulin as a healthcare supplement for GDM patients. Further studies could include analysis of cellular energy metabolism and other pathways in mediating the anti-diabetic actions of inulin.

## Supplementary Information

**Additional file 1:** The original bands of Western Blot Analysis.

## Data Availability

The datasets used and/or analyzed during the current study are available from the corresponding author upon reasonable request.

## Abbreviations

GDM: Gestational diabetes mellitus
SCFAs: Short-chain fatty acids
NCD: Normal chow diet
HFD: High-fat/sucrose diet
FBG: Fasting blood glucose
IR: Insulin resistance
AGEs: Advanced glycation end products
OGTT: Oral glucose tolerance test
AUC: Area under the curve
TG: Triglycerides
TC: Total cholesterol
LDL: Low-density lipoprotein
HDL: High-density lipoprotein
RETN: Resistin
IRS: Insulin receptor
Akt: Protein kinase B
ERK 1/2: Extracellular signal-regulated protein kinases 1 and 2
GLUT4: Glucose transporter isoform 4
