# Supplementary material for: Dietary supplementation of inulin alleviates metabolism disorders in gestational diabetes mellitus mice via RENT/AKT/IRS/GLUT4 pathway
Source: Diabetol Metab Syndr. 2021 Dec 24;13:150. doi: 10.1186/s13098-021-00768-8 (PMC8709963; doi:10.1186/s13098-021-00768-8)
Supplement: Supplementary file 1 — Additional file 1: The original bands of Western Blot Analysis. [file 13098_2021_768_MOESM1_ESM.docx]

We are sorry about not providing the original uncropped full blots, because all the blots were cropped into different bands which were between different fractions as follows (Figure 2-4). We added this information in the supplement materials.


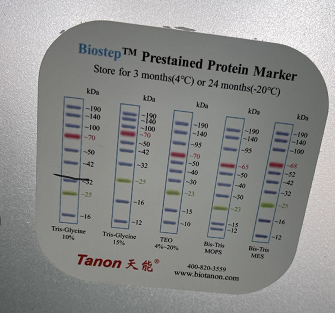


**Figure 1 The information of marker we used.**

**
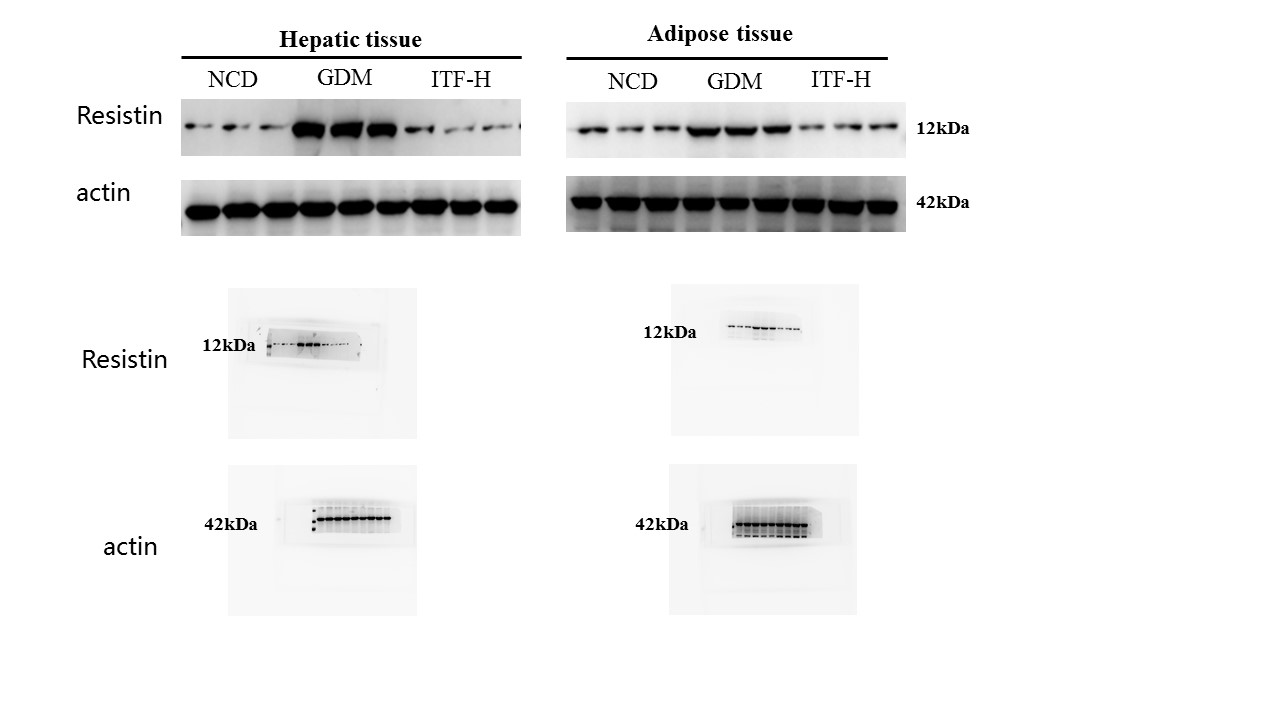
Figue 2 Changes of hepatic and adipose RETN protein expression levels.** The blot of“Actin”with molecular mass of 42 kda was cropped into a band between fractions 25 and 70 kda; The blot of“Resistin”with molecular mass of 12 kda was cropped into a band between fractions loadingbuffer and 25 kda.


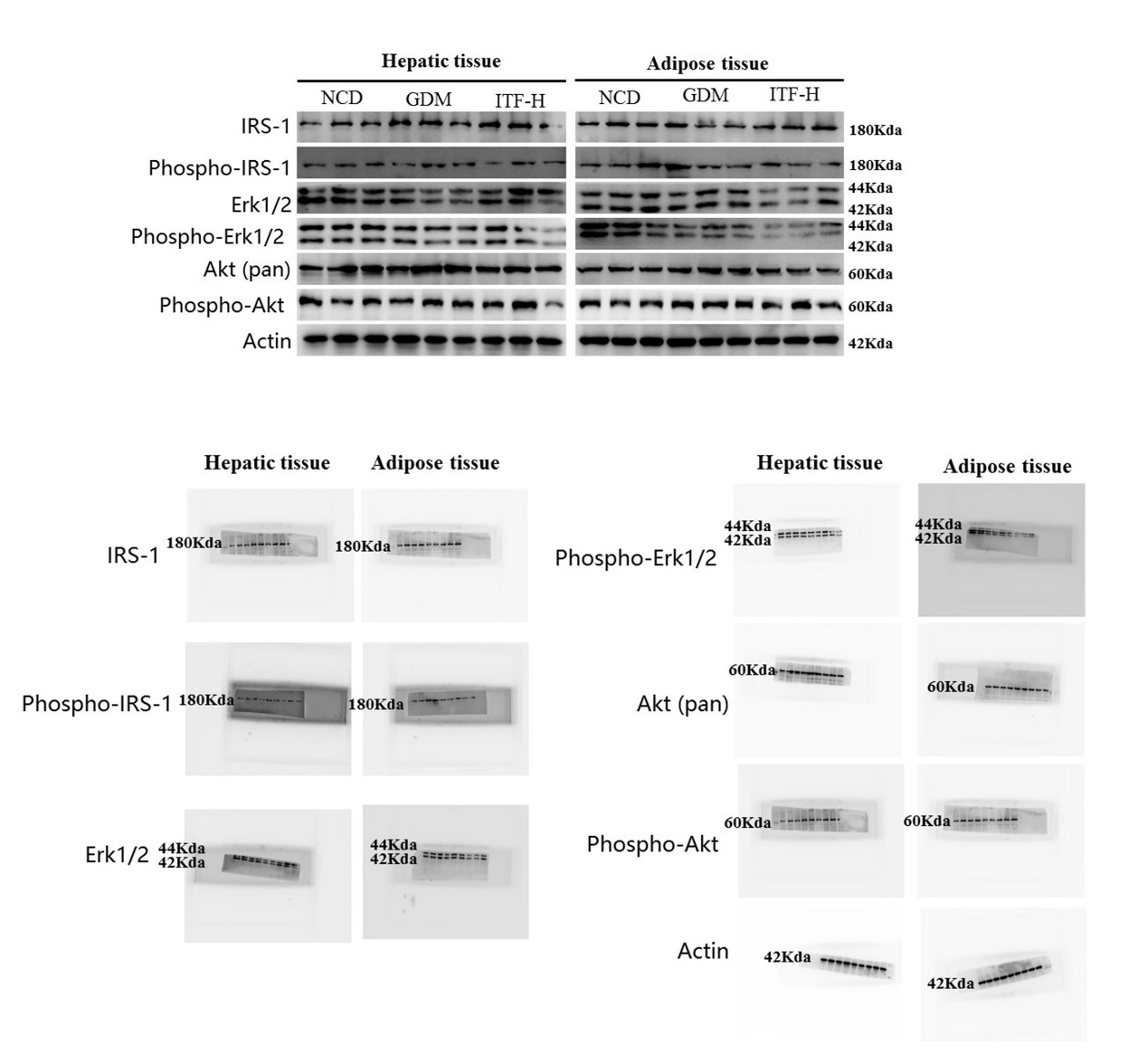


**Figure 3 Changes of hepatic and adipose IRS-1, Erk and Akt protein expression levels.** The blot of“Actin”with molecular mass of 42 kda was cropped into a band between fractions 25 and 70 kda; The blot of“IRS-1”with molecular mass of 180 kda was cropped into a band between fractions 70 and 190 kda; The blot of“p-IRS-1”with molecular mass of 180 kda was cropped into a band between fractions 70 and 190 kda; The blot of“ERK1/2”with molecular mass of 44/42 kda was cropped into a band between fractions 25 and 50 kda; The blot of“p-ERK1/2”with molecular mass of 44/42 kda was cropped into a band between fractions 25 and 50 kda; The blot of“AKT”with molecular mass of 60 kda was cropped into a band between fractions 42 and 100 kda; The blot of“p-AKT”with molecular mass of 60 kda was cropped into a band between fractions 42 and 100 kda.


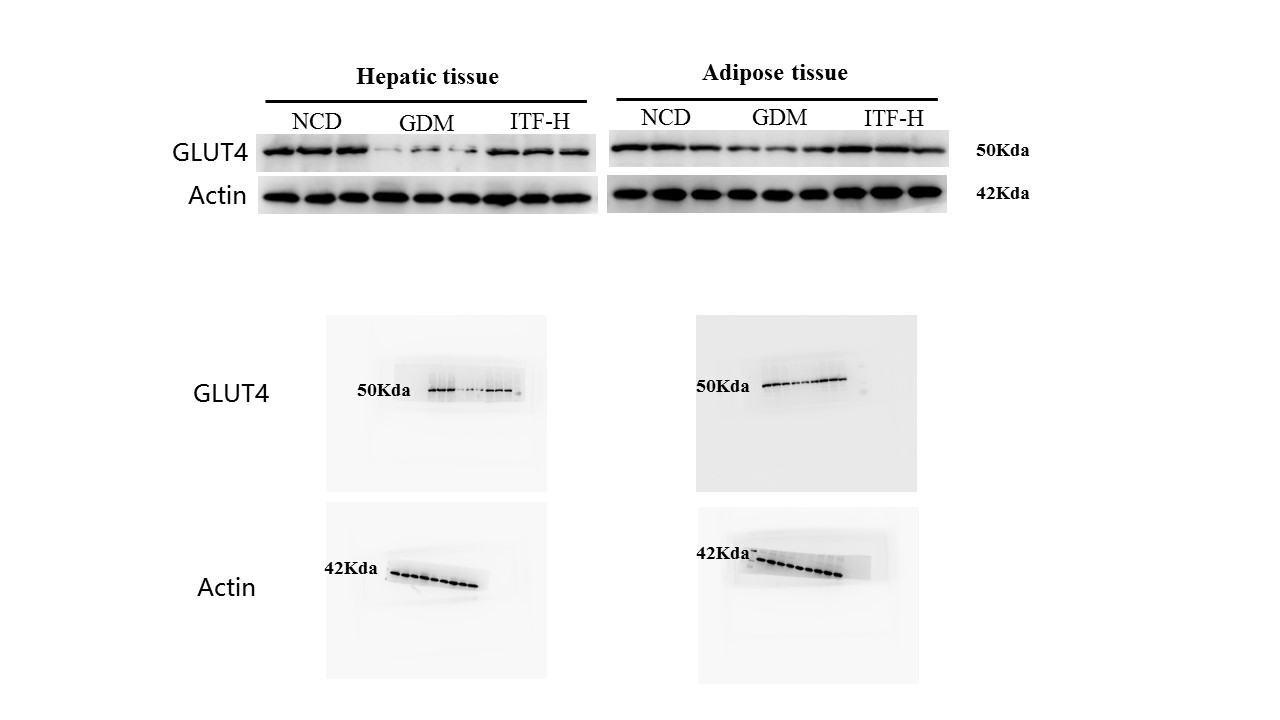


**Figure 4 Changes of hepatic and adipose GLUT4 protein expression levels.**

The blot of“Actin”with molecular mass of 42 kda was cropped into a band between fractions 25 and 70 kda; The blot of“GLUT4”with molecular mass of 50 kda was cropped into a band between fractions 32 and 100 kda.
